# Impact of 3D-Printed Tricalcium Phosphate Scaffold Polymorphism and Post-Processing Variations on Bone Regenerative Outcomes

**DOI:** 10.3390/bioengineering13010034

**Published:** 2025-12-28

**Authors:** Nicholas Jose Iglesias, Sara E. Munkwitz, Hana Shah, Savanah R. Sturm, Nicholas A. Mirsky, Adriana I. Sandino, Ricky Almada, Vasudev Vivekanand Nayak, Lukasz Witek, Paulo G. Coelho

**Affiliations:** 1 DeWitt Daughtry Family Department of Surgery, University of Miami Miller School of Medicine, Miami, FL 33136, USA; 2 University of Miami Miller School of Medicine, Miami, FL 33136, USA; 3 Florida International University Herbert Wertheim College of Medicine, Miami, FL 33199, USA; 4 Department of Biochemistry and Molecular Biology, University of Miami Miller School of Medicine, Miami, FL 33136, USA; 5 Dr. John T. Macdonald Foundation Biomedical Nanotechnology Institute (BioNIUM), University of Miami, Miami, FL 33136, USA; 6 Biomaterials and Regenerative Biology Division, NYU College of Dentistry, New York, NY 10010, USA; 7 Department of Biomedical Engineering, NYU Tandon School of Engineering, Brooklyn, NY 11201, USA; 8 Hansjörg Wyss Department of Plastic Surgery, NYU Grossman School of Medicine, New York, NY 10016, USA; 9 Department of Oral and Maxillofacial Surgery, NYU College of Dentistry, New York, NY 10010, USA; 10 DeWitt Daughtry Family Department of Surgery, Division of Plastic Surgery, University of Miami Miller School of Medicine, Miami, FL 33136, USA; 11 Sylvester Comprehensive Cancer Center, University of Miami Miller School of Medicine, Miami, FL 33136, USA

**Keywords:** calcium phosphate, hydrothermal immersion processing, sintering, direct inkjet writing, bone scaffold, 3D printing

## Abstract

Tricalcium phosphate (TCP) bioceramics, available as α- and β-polymorphs, are frequently employed in the production of three-dimensionally (3D) printed bone scaffolds. Although hydrothermal immersion processing (HP) and sintering (S) are commonly adopted as post-printing techniques for bioceramics, a comprehensive comparative analysis of their effects on the osteogenic performance of α- and β-polymorphs in vivo remains inadequately investigated. In this study, α-TCP and β-TCP scaffolds were fabricated via direct ink writing and subjected to hydrothermal immersion processing (α-TCP/HP) and sintering (β-TCP/S) prior to implantation in *n* = 12 skeletally mature sheep (*n* = 1 scaffold per group per animal), and the outcome variables were evaluated at 3 and 12 weeks postoperatively (*n* = 6 sheep per time point). The quantitative results showed no significant differences in bone deposition or scaffold resorption at 3 weeks postoperatively (*p* = 0.618 and *p* = 0.898, respectively). However, at 12 weeks, there was a significant increase in osteogenesis and scaffold resorption in the β-TCP/S cohort relative to the α-TCP/HP counterparts (*p* < 0.001 and *p* = 0.004, respectively). β-TCP scaffolds subjected to post-print sintering exhibited superior osteoconductive and resorptive profiles compared to hydrothermal immersion-processed α-TCP scaffolds over the 12-week healing period.

## 1. Introduction

Large bone defects have traditionally been managed with either autografts or allografts [1]. While autografts are considered to be the gold standard, they are associated with donor site morbidity and limited supply, whereas allografts increase the patient’s risk of infection and graft rejection [2]. To reduce these risks, calcium phosphate (CaP)-based bioceramic scaffolds have emerged as a transformative solution by offering chemical similarity to native bone mineral composition and osteoconductive potential [3,4]. Computer-aided design (CAD) and additive manufacturing have further transformed this field by enabling the rapid fabrication of patient-specific CaP scaffolds. Specifically, scaffolds may be customized with controlled pore size and interconnectivity that have been shown to restore form and function at the bony defect site [3,5].

Among CaP-based bioceramics, tricalcium phosphate [TCP, Ca_3_(PO_4_)_2_] is frequently utilized due to a favorable safety profile [6,7,8,9]. Among its three polymorphs (α, α′, and β), the β-polymorph is stable at room temperature until ~1120 °C, wherein it transforms to α-TCP (between 1120 °C and 1430 °C) and remains in a metastable phase [10]. Alternatively, room temperature-stable α-TCP can be produced via thermal crystallization of amorphous precursors above the transformation temperature [11]. Above 1430 °C, the α′-TCP metaphase emerges, though it cannot exist at room temperature [10]. The difference in biological activity between the two (α and β) polymorphs may have direct implications toward their use in tissue-engineering strategies. Ultimately, in addition to the local microenvironmental factors, including temperature, pH, and processing techniques, the biomechanical and bioactive properties of either polymorph are dependent on the post-processing steps utilized in fabricating the scaffold end product [6,11,12,13,14,15].

The post-processing method used can influence phase composition, nanostructure, mechanical behavior, and osteogenic responses in vivo [16]. Of note, the hydrothermal immersion processing of α-TCP promotes the formation of nano structural calcium-deficient hydroxyapatite while preserving the scaffold’s architectural integrity [16]. Building on this, previous work has also focused on evaluating the physicochemical properties of three-dimensionally (3D) printed CaP scaffolds and their post-printing workflows [16,17]. Alternatively, sintering of β-TCP has been shown to cause necking and subsequent fusion of ceramic particles, resulting in the evaporation of toxic polymeric binders/additives and an increase in the mechanical strength of the end product [18,19]. These two most commonly utilized bioceramic polymorphs and post-processing techniques can translate into distinct dissolution and osteoconductive responses in vivo, thus potentiating direct implications for their use in tissue-engineering strategies.

Given the central role of processing in scaffold performance, there is a need to directly compare the osteoconductive and regenerative behavior between α-TCP and β-TCP constructs post-processed through hydrothermal immersion- and sintering-based routes. This study aimed to evaluate the osteoconductivity, scaffold resorption, and bone regeneration associated with the aforementioned bioceramic scaffolds in a challenging low-bone-density environment in a large translational ovine model.

## 2. Materials and Methods

### 2.1. Scaffold Synthesis

Two cylindrical scaffold designs were used in this study. The control scaffold (α-TCP/HP—MimetikOss^®^ 3D/creos^TM^ syntogain 3D, Mimetis Biomaterials S.L., Barcelona, Spain) was first designed (Solidworks, Dassault Systems, Vélizy-Villacoublay, France) to be 6 mm in diameter and 6 mm in height with an orthogonal lattice pattern. α-TCP powder was mixed with a 30 wt./vol. poloxamer-407 aqueous binding solution at a liquid-to-solid ratio of 0.45 wt./wt. The α-TCP powder was synthesized by mixing calcium carbonate (Sigma-Aldrich, Darmstadt, Germany) and monetite (Sigma-Aldrich, Darmstadt, Germany) in a furnace (Hobersal CNR-58, Barcelona, Spain) at 1400 °C with immediate quenching to stabilize the TCP powder α phase [20]. Using a nozzle size of 250 µm, a layer height of 225 µm, an infill density of 45%, and a deposition speed of 10 mm/s, a custom-made direct ink writing (DIW) printer (Heavy-Duty Paste Extruder, CIM-UPC, Barcelona, Spain) was used to print the scaffolds at ambient temperature [16]. The printed scaffolds were then subjected to a hydrothermal immersion process that included immersion in water and autoclaving at 121 °C for 30 min.

The experimental scaffolds (β-TCP/S) were also designed using CAD (RoboCAD v6, 3D Inks LLC, Tulsa, OK, USA) with the same dimensions as mentioned above. The TCP powder (Sigma-Aldrich, Germany) was first calcined at 800 °C for 11 h, followed by attrition milling in distilled water and drying in a low-temperature oven (~37 °C) [19]. The dried β-TCP ceramic powder was then used to prepare a colloidal gel (with a ~0.43 wt./wt. solid-to-liquid ratio) by the addition of distilled water, ammonium polyacrylate, hydroxypropyl methylcellulose, and poly(ethylenimine), as shown previously [19]. The resulting colloidal gel was loaded into Luer-lock syringes of a custom-built DIW printer (3D Inks LLC, Tulsa, OK, USA). The β-TCP/S scaffolds were then printed with similar settings as mentioned above at ambient temperature under a low-viscosity paraffin oil bath. The printed scaffolds were allowed to dry at 60 °C for 11 h and sintered at 1100 °C for 4 h. The scaffolds were then autoclaved at 121 °C for 30 min.

### 2.2. Surgical Procedure

**Figure 1:**
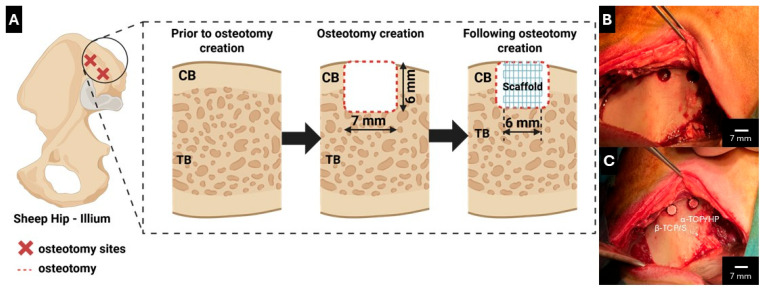
(**A**) Schematic illustration of osteotomy positioning on sheep iliac crest within cortical bone (CB) and trabecular bone (TB). Figure generated on Biorender.com. Surgical aspect of the osteotomy sites (**B**) pre- and (**C**) post-scaffold implantation.

All procedures were reviewed and approved by the Institutional Animal Care and Use Committee of École Nationale Vétérinaire d’Alfort (Maisons-Alfort, Ile-de-France, France). *n* = 12 adult sheep were allowed to acclimate for ~1 week prior to any surgical intervention. General anesthesia was induced using sodium pentothal (15–20 mg/kg) in Normasol solution via injection into the jugular vein. Anesthesia was maintained using isoflurane (1.5–3%) in O_2_/N_2_O (50/50). The animals were monitored throughout the procedure using a combination of pulse oximetry, wave form capnography, and electrocardiography. The sheep ilium was then shaved, sterilized, and draped in standard fashion. A 10 cm anteroposterior incision was made overlying the ilium, exposing the iliac bone. Two osteotomies were made per animal (7 mm in diameter and 6 mm in depth) via rotary instrumentation under continuous saline irrigation. The osteotomies received either an α-TCP/HP or a β-TCP/S scaffold (Figure 1), and the animals were randomized to heal for either 3 or 12 weeks (*n* = 6 animals/time point). Incisions were closed in layers using absorbable sutures and 2-0 nylon for skin. Postoperatively, antibiotic prophylaxis (cefazolin, 500 mg) was administered intravenously. At predefined timepoints (at either 3 or 12 weeks), the animals were euthanized and iliac bones were harvested en bloc.

### 2.3. Microcomputed Tomography (µCT) and Volumetric Reconstruction

At the predefined endpoints, samples were stored in a 10% neutral buffered formalin and later transferred to 70% ethanol (EtOH) solution prior to µCT (µCT 40, Scanco Medical, Basserdorf, Switzerland). The µCT was set to a resolution of 18 µm at 70 kVp and 114 µA. Digital Communication in Medicine (DICOM) scan files were imported into Amira (v.6.3.2, ThermoFischer Scientific, Waltham, MA, USA) for reconstruction and qualitative visualization of bone ingrowth and scaffold architecture. Regions of interest (ROIs) were manually segmented using Hounsfield Units (HUs) of bone and scaffold by a single trained and calibrated user.

### 2.4. Histological Processing and Analysis

After completion of µCT imaging, the samples were further dehydrated in a series of EtOH solutions of increasing concentrations (70–100% *v*/*v*) over a 96 h period. Upon completion of the dehydration process, the samples were immersed in methyl salicylate for 48 h and embedded in a methacrylate-based resin. The embedded samples were cut into ~300 µm-thick sections using a low-speed precision wafering saw (Isomet 2000, Buehler Ltd., Lake Bluff, IL, USA) and glued to slides using a low-viscosity cyanoacrylate-based adhesive (Loctite 408, Henkel AG & Co. KGaA, Düsseldorf, Germany). The slides were ground to a final thickness of ~100 μm with progressively finer abrasive papers (400, 600, 800, and 1200 grit) on a grinding machine (Metaserv 3000, Buehler, Lake Bluff, IL, USA) with abundant irrigation. Each slide was polished with an alumina-based solution (1 μm MicroPolish^TM^, Buehler, Lake Bluff, IL, USA) on a micro-fiber cloth for 2 min [21]. Slides were stained with Stevenel’s Blue and Van Gieson picro-fuschin (SVG) and digitally scanned (Aperio CS2, Leica, Wetzlar, Germany) for quantitative histomorphometric analysis.

For the quantitative histomorphometric analysis, bone (bone %), scaffold (scaffold %), and soft tissue/empty space (soft tissue/empty space %) within the ROI were manually segmented using image processing software (Photoshop 2024, Adobe Systems Incorporated, San Jose, CA, USA). Image analysis was performed (JV Analysis 2014; NYU Biomaterials and Regenerative Biology Division, New York, NY, USA; USA/UM Miller School of Medicine, Department of Biochemistry and Molecular Biology, Miami, FL, USA) to determine the percentage areas of the ROI occupied by the bone, scaffold, and soft tissue/empty space. Soft tissue/empty space was calculated by subtracting the percentage area occupied by the scaffold and bone from the total area of the ROI. The ROI included the area encompassed within the borders of the 6 mm × 6 mm scaffold on a 2-dimensional histomicrograph (*n* = 1 slice per defect) obtained from the defect center.

### 2.5. Statistical Analysis

Quantitative histomorphometric data was assessed using a general linear mixed-model analysis of variance and a least-significant difference (LSD) post hoc analysis with time and scaffold groups set as fixed factors. Statistical analyses were performed using SPSS (v31, IBM Corp., Armonk, NY, USA). All values were reported as means ± 95% confidence intervals (95% CI) unless otherwise specified. A *p* value < 0.05 was considered statistically significant.

## 3. Results

### 3.1. Microcomputed Tomography (µCT) Volumetric Reconstruction

**Figure 2:**
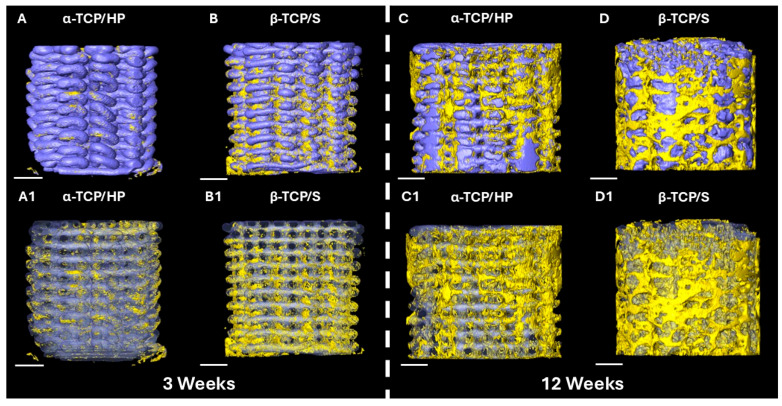
Representative volumetric reconstruction images of scaffolds within osteotomies after 3 (**A**,**B**,**A1**,**B1**) and 12 weeks (**C**,**D**,**C1**,**D1**) postoperatively. Yellow layers denote bone and purple layers highlight scaffold presence. (**A**–**D**) Bone and scaffold layers in 100% opacity. (**A1**–**D1**) Scaffold opacity of 50% and bone opacity of 100% to aid in visualization. Scale bars are 1 mm in length.

Qualitative 3D analysis of the µCT volumetric reconstruction at 3 weeks demonstrated a relative increase in bony deposition along the periphery of the β-TCP/S scaffolds at the osteotomy margins relative to α-TCP/HP (Figure 2A,B,A1,B1). Both scaffold structures remained intact, with minimal resorption observed on 3D imaging. At 12-weeks, the β-TCP/S scaffold group presented increased bone formation along the periphery and within the center of the scaffold architecture compared to α-TCP/HP. While both scaffold groups began to undergo resorption by 12 weeks, there was a greater qualitative reduction in the β-TCP/S scaffold presence relative to the α-TCP/HP counterparts (Figure 2C,D,C1,D1).

### 3.2. Qualitative Histological Findings

**Figure 3:**
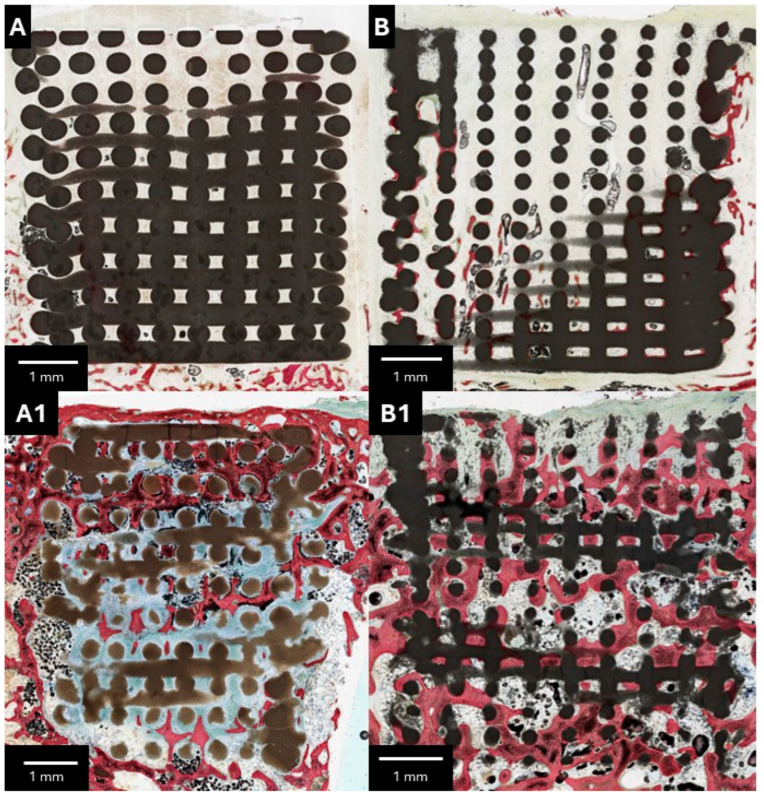
Representative low-magnification histomicrographs of (**A**,**A1**) α-TCP/HP and (**B**,**B1**) β-TCP/S scaffolds at (**A**,**B**) 3 and (**A1**,**B1**) 12 weeks postoperatively. SVG stained bone in red, soft tissue/inflammatory infiltrate in blue, while black/gray regions represent the scaffolds.

**Figure 4:**
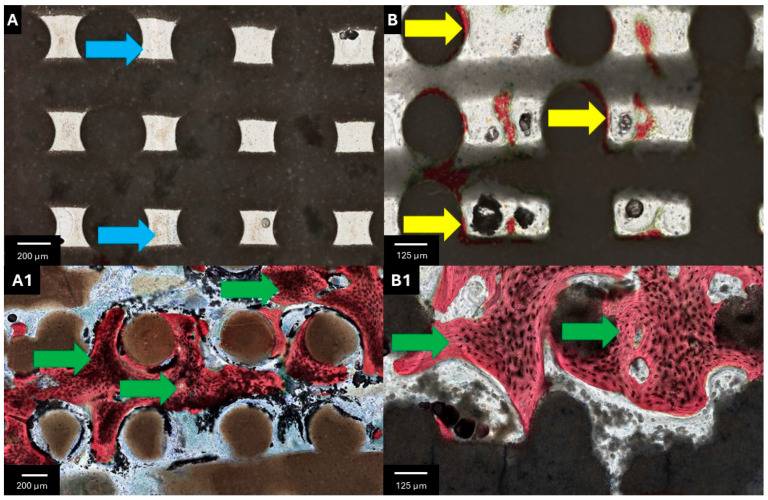
Representative high-magnification histomicrographs of (**A**,**A1**) α-TCP/HP and (**B**,**B1**) β-TCP/S scaffolds at (**A**,**B**) 3 and (**A1**,**B1**) 12 weeks postoperatively. Blue arrows depict areas devoid of bone within the scaffold lattice architecture, yellow arrows depict early bone formation in direct contact with the scaffold, and green arrows highlight areas of regenerating woven and lamellar bone formation. SVG stained bone in red, soft tissue/inflammatory infiltrate in blue, while black/gray regions represent the scaffolds.

Upon gross dissection, no evidence of adverse tissue response was noted. At 3 weeks, while the β-TCP/S scaffold demonstrated bone regeneration along the peripheral regions, both cohorts were largely devoid of osteogenesis at the scaffold center (Figure 3A,B). At higher magnification, the α-TCP/HP scaffolds revealed an increased prevalence of inflammatory cells within the strut interstices, with minimal bone deposition at the periphery (Figure 4A,B). At 12 weeks, increased osteogenesis was observed throughout the scaffold architecture. While the α-TCP/HP scaffold revealed lower resorption and osteogenesis relative to the β-TCP/S (Figure 3A1,B1), both scaffolds stimulated the formation of mature, woven bone (Figure 4A1,B1). Subjectively, the α-TCP/HP scaffolds presented elevated levels of soft tissue/empty space within the strut interstices compared to the β-TCP/S counterparts. However, consistent with gross anatomic evaluation, no foreign body reaction to either scaffold design was observed.

### 3.3. Histomorphometric Findings

**Figure 5:**
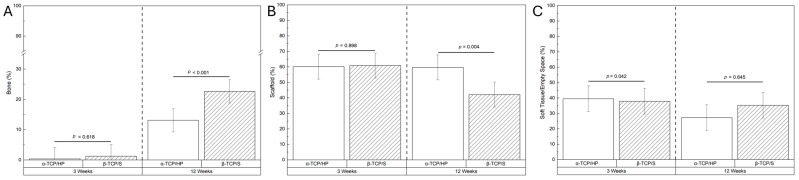
Histomorphometric analysis of (**A**) new bone formation, (**B**) scaffold presence, and (**C**) soft tissue/empty space within the ROI.

In quantitative histomorphometric analysis, no significant differences in new bone formation or scaffold resorption were observed at 3 weeks (Figure 5A,B). At 12 weeks, there was a statistically significant increase in osteogenesis and scaffold resorption in the β-TCP/S group relative to the α-TCP/HP counterparts (*p* < 0.001 and *p* = 0.004, respectively) (Figure 5A,B). Three weeks postoperatively, there was a statistically significant decrease in soft tissue/empty space in the β-TCP/S cohort (*p* = 0.042), but this difference was not observed at the 12-week timepoint (Figure 5C).

## 4. Discussion

Both scaffold designs used in this study incorporated a lattice structure wherein pores between the struts served as healing chambers that promoted directional osseoconduction [22]. Overall, the results demonstrate that β-TCP/S supported significantly greater osteogenesis and scaffold resorption at 12 weeks compared to α-TCP/HP scaffolds. Qualitative µCT and histological analyses further revealed enhanced bone ingrowth and remodeling within the β-TCP/S group. These findings highlight the critical role of material selection and processing in dictating scaffold functionality and biological outcomes.

Ideally, a scaffold should degrade alongside new tissue formation to maintain mechanical integrity early on while gradually creating space for mineralized bone during advanced stages of healing. Although α-TCP has been shown to generally exhibit faster hydrolysis compared to β-TCP in in vitro settings, this does not necessarily reflect the functional degradation of a scaffold in vivo [23]. Hydrothermal immersion processing of an α-TCP scaffold accelerates the hydrolysis of α-TCP to calcium-deficient hydroxyapatite (CDHA) [24]. This process results in a remodeling of CDHA into a needle-like morphology [16,25]. This needle-like morphology may not majorly impact the macro- or microporosity of the 3D-printed scaffolds. Moreover, prior studies on hydrothermal-immersed α-TCP scaffolds have reported ultimate flexural and compressive strengths of 1.38 MPa and 4.61 MPa, respectively [26]. In contrast, sintering the β-TCP/S scaffolds has numerous structural and bioactive implications that could help explain the increased rates of osteogenesis and resorption reported in this study. Scanning electron micrographs comparing “green” (pre-sintered) and sintered β-TCP/S scaffolds have shown that sintering fuses ceramic particles via necking, resulting in an overall reduction in microporosity [18,19]. These microstructural changes translate into higher flexural, tensile, and compressive strength, as well as an increased Young’s modulus [14,19], with prior literature reporting ultimate flexural and tensile compressive strengths of 6.6 and 9.6 MPa, respectively [19].

Importantly, these mechanical properties have direct effects on the resulting biological activity. Scaffolds tuned to match the mechanical behavior of native bone elicit superior biological responses, including increased cell proliferation, deeper cellular infiltration, and enhanced osteogenic differentiation [9,18]. Such engineered scaffold architectures also permit physiological loading and remodeling, ultimately restoring form and function at the defect site [18,27]. This relationship between mechanical competence, load bearing, and cell-mediated remodeling may explain why the β-TCP/S scaffolds exhibited greater resorption (~30%) and significantly higher bone volume at 12 weeks despite β-TCP’s nominally slower in vitro dissolution rates. These results align with findings from a long-term translational study by Shen et al. wherein β-TCP scaffolds placed in an alveolar bone defect model underwent > 99% resorption by 18 months, corresponding to a ~90.5% annual degradation rate [9]. These findings by Shen et al. highlighted that β-TCP can undergo rapid remodeling when mechanically engaged in a defect microenvironment. Although the present study does not provide immunohistochemical evidence of the mechanisms driving bone formation and scaffold resorption, results indicate that β-TCP/S scaffolds degrade faster, which may be attributed to a better balance between their mechanical properties and cell-driven remodeling. In contrast, the α-TCP/HP scaffolds may have degraded at a slower rate due to lower mechanical strength and associated attenuation of the biological activity necessary to elicit resorption, necessitating follow-up evaluation.

Beyond the biomechanical implications of scaffold structures, the microstructure of TCP scaffolds has direct biological effects. Specifically, the microstructure favors cellular adhesion and proliferation of stem cells to promote osteogenesis. Porous TCP scaffolds provide a microenvironment wherein stem cells may attach on the surface and within pore spaces as early as 24 h after implantation [28,29]. Consequently, the stem cells survive, proliferate, and ultimately differentiate on the TCP granules [28]. The underlying mechanism by which β-TCP scaffolds promote osteogenesis is upregulation of genes such as Runt-related transcription factor 2, Secreted Phosphoprotein 1, and Bone Gamma-Carboxyglutamic Acid Protein, which promote stem cell differentiation and osteoblast proliferation [29,30]. Furthermore, the TCP scaffold may further reduce the inflammation and fibrotic remodeling that occurs in large defects, directing tissue regrowth toward osteogenesis [30]. Ultimately, the microstructure inherent within TCP scaffolds works to promote osteogenesis in osseous defects through a variety of cellular mechanisms.

On a separate note, scaffold degradation has been shown to be slower in cortical environments compared to trabecular bone due to differences in native biomechanical properties [4,9]. Relative to cortical bone, trabecular bone has an increased porosity, vascularity, and surface area [31], which permits rapid cell and nutrient transport [32]. The increased bone turnover rate and vascularity within trabecular bone result in a more metabolically active microenvironment, facilitating rapid scaffold degradation [9,31,32]. These differences highlight the importance of considering the local microenvironment when designing scaffolds, as material resorption should closely match the rate of osteogenesis, warranting future evaluation of these scaffolds in cortical bone environments [33,34].

While the presented study reports the biocompatibility and osteoconductive differences between α-TCP/HP and β-TCP scaffolds, there remain several limitations. As this is a single-site model, the bioactive and osteoconductive effects of these scaffold designs were only analyzed in one microenvironment—the ovine ilium. As aforementioned, microenvironmental differences exist between the cortical and trabecular bone, which warrants follow-up evaluation in other defect sites such as the mandible (primarily dense Type-I bone). Furthermore, while the 3D imaging and histological evaluation of scaffold resorption and bone regeneration is robust, additional molecular analyses may better describe the mechanistic implications of the bioceramics utilized for scaffold synthesis and corresponding post-processing techniques.

Despite the promising translational data, process standardization, quality control, and regulatory considerations remain barriers to clinical application [35,36]. Regulatory oversight encompasses the print and post-processing workflow, making both sintering and hydrothermal immersion subject to regulatory review and approval prior to clinical use [36]. Notably, because hydrothermal immersion processing involves the use of an autoclave, a routinely used sterilization technique, it might ultimately be deemed substantially equivalent to current standards [26]. Looking forward, continued improvement of manufacturing workflows, scaling of production methods, and refinement of regulatory pathways will be essential to facilitate clinical translation.

## Data Availability

The raw data supporting the conclusions of this article will be made available by the authors on request.
